# Supplementary material for: Remote Effect of Insecticide-Treated Nets and the Personal Protection against Malaria Mosquito Bites
Source: PLoS One. 2017 Jan 27;12(1):e0170732. doi: 10.1371/journal.pone.0170732 (PMC5271322; doi:10.1371/journal.pone.0170732)
Supplement: S1 Text — (DOCX) [file pone.0170732.s001.docx]

# Summary of the WHOPES Experimental Hut trial protocol (from reference (1))

**Figure 1: Design of the experimental huts commonly used in West Africa.**

Experimental stations are composed of several identical experimental huts designed to resemble local housing. Huts of the typical West African style are made from concrete bricks with a corrugated iron roof, a ceiling of polythene sheeting and a concrete base surrounded by a water-filled moat to prevent the entry of ants. Mosquitoes can enter the huts through four window slits. The design of the window slits allows easy entry but greatly limits the egress of mosquitoes once they have entered the hut. Baseline information should be obtained on the attractiveness of the huts and the recapture rates of live and dead mosquitoes released in the huts. Contact bioassays should be conducted on the walls to rule out contamination that could affect the outcome of the study.

Candidate LNs are compared with a negative control (an untreated net of the same or similar netting material, denier and mesh size) and a positive control (a WHOPES-recommended LN with the same or similar specifications unwashed and washed 20 times or a CTN washed just before exhaustion).

Each week, the treatment arms are rotated among the huts according to a Latin square scheme. Several replicates of each treated net are needed to rotate each day during one week and reused the next weeks following the same rotation. At the end of the week, the huts are carefully cleaned and aired to remove potential contamination. Before use in the study, each net (including control) should have a total of six holes (4 cm x 4 cm) cut in the sides using sharp scissors to simulate the conditions of a torn net (two holes on each large side and one on each small side).

Adult volunteers sleep under the nets, and mosquitoes are collected the next morning. Informed consent should be obtained from all volunteers participating in the study. Effective chemoprophylaxis should be provided where appropriate, and volunteers should be medically supervised. Sleepers are rotated randomly among huts each night of the study. They shall enter the hut at dusk and remain inside until dawn. In the morning, dead mosquitoes are collected from the floor of the hut as well as from the verandah trap and inside the nets; resting mosquitoes are collected using aspirators from inside the net and from the walls and roof of the hut and verandah trap. Mosquitoes are scored by location as dead or alive and as fed or unfed. Live mosquitoes should be placed in small cups and provided with access to sugar solution for 24 hours to assess delayed mortality.

## References:

1. WHO. Guidelines for laboratory and field-testing of long-lasting insecticidal nets. World Health Organization; 2013 p. 89. Report No.: WHO/HTM/NTD/WHOPES/2013.3.
